# Electrochemical Characterization of Recovered Lead from Lead–Acid Battery Recycling Using Wet/Melt Quenching Method

**DOI:** 10.3390/ma19153311

**Published:** 2026-08-04

**Authors:** Delia Niculina Piscoiu, Simona Rada, Tudor Panfil Toader, Horatiu Vermesan

**Affiliations:** 1 Faculty of Materials and Environmental Engineering, Technical University of Cluj-Napoca, 400641 Cluj-Napoca, Romania; deliapiscoiu95@gmail.com (D.N.P.); tudor.toader@incerc-cluj.ro (T.P.T.); horatiu.vermesan@imadd.utcluj.ro (H.V.); 2 National Institute for Research and Development of Isotopic and Molecular Technologies, 400293 Cluj-Napoca, Romania; 3 National Institute for Research and Development in Urban Planning and Sustainable Territorial Development, 400524 Cluj-Napoca, Romania

**Keywords:** wet method, melt quenching method, X-ray diffraction (XRD), electrochemical impedance spectroscopy (EIS), cyclic voltammetry (CV), linear sweep voltammetry (LSV)

## Abstract

In recent years, alternative recycling approaches such as melt quenching and electrochemical evaluation methods have been investigated to assess the quality and performance of recovered lead materials. In this study, several samples obtained from the recycling process were analyzed electrochemically. The objective was to compare their electrochemical parameters and identify the samples with better electrochemical performance. The main methods used in this paper are X-ray diffraction analysis and voltammetric investigations using cyclic voltammetry (CV), linear sweep voltammetry (LSV), and electrochemical impedance spectroscopy (EIS). Electrochemical characterization provides valuable information about the behavior of recycled lead materials. Parameters such as half-wave potential (E_1/2_), anodic current density (I_a_), and solution or bulk resistance (R_b_) are commonly used to evaluate electrochemical activity and conductivity. The electrochemical analysis reveals noticeable differences among the samples studied. The analysis of CV, LSV, and EIS indicates that the samples P2 (doped with CuO and Sb_2_O_3_) and P3N (doped with CaO/Fe_2_O_3_/Fe) exhibit the most favorable electrochemical behavior for lead acid battery applications, with the highest current response and smallest peak separation, suggesting efficient Pb/PbSO_4_ redox reactions and minimal polarization.

## 1. Introduction

Recycling materials from lead–acid batteries is important because they contain large amounts of recoverable lead and are widely used in automotive and industrial applications. The development of efficient waste management strategies is closely linked to the recycling and reuse of spent automotive batteries. From an electrochemical perspective, extending battery rechargeability over multiple cycles remains a key objective. The recycling of batteries plays a crucial role not only in conserving natural resources but also in reducing the environmental impact.

Currently, increasingly stringent requirements are being imposed on automotive batteries, including higher energy density and power, improved safety, extended charge–discharge cycle life, operation over a wide temperature range, and reduced production costs. Achieving performance levels comparable to those of fuel-based vehicles while meeting market expectations remains a major challenge.

Although numerous techniques for lead recycling have been proposed, many suffer from significant drawbacks, including process complexity and toxicity, low solubility of lead-based compounds in common solvents, inefficient desulfatization, and difficulty reconverting these compounds into electrochemically active metal oxides.

Therefore, the efficient management of hazardous battery waste and the recovery of valuable materials represent a considerable challenge. Nevertheless, the recycling of spent automotive batteries provides a valuable secondary source of raw materials and contributes to the development of a stable and sustainable supply chain.

Recent studies indicate that the doping of the recycled lead with appropriate amounts of CaO, CuO, or Fe has a beneficial effect on the electrochemical properties of the material [1]. For example, the hydrogen evolution reactions no longer occur, cyclic voltammograms indicate good reversibility, and the electrochemical properties are improved [2,3,4,5].

By doping Pb-PbO_2_-based glass-ceramics extracted from sulfated anodic and cathodic plates of a spent automotive battery with calcium oxide [6], XRD data indicate that sulfate and sulfite ions are immobilized within the glassy network due to their affinity for calcium ions. The presence of iron ions in these lead- and calcium oxide-based glass-ceramics indicates a partial desulfatization of the spent plates, leading to the partial conversion of lead sulfate phases into lead dioxide and metallic lead [3]. The obtained materials can be used as new electrodes in lead–acid batteries, which is promising from a practical standpoint [2].

In recent years, the incorporation of calcium ions into lead electrodes has yielded materials with enhanced electrochemical performance [7], including increased energy density, reduced costs, and extended cycle life [8,9,10,11]. According to the literature [12], the use of lead–calcium batteries in automotive applications offers clear advantages, including overcharge tolerance, high-temperature operation, reduced water consumption, and lower self-discharge rates.

Copper oxide (CuO) is of particular interest due to its wide range of applications in photovoltaics, biosensors, and medicine. CuO nanoparticles have demonstrated significant antibacterial activity against a broad spectrum of microorganisms, acting as antimicrobial and antioxidant agents, and showing promising potential in anticancer therapy [13,14]. Recent studies have also reported the use of CuO in gas sensors [15] and in thin-film solar cell technologies [16].

To enable the use of recycled and doped materials as active electrodes in lead–acid batteries, a detailed investigation of their structural and electrochemical properties is required. In this context, materials doped with calcium oxide, iron oxide, and/or iron were systematically analyzed. This approach contributes to sustainable development by conserving natural resources and promoting innovative pathways for secondary lead production.

Iron oxide (Fe_2_O_3_) plays a key role in enhancing battery performance by facilitating more efficient charge–discharge processes and mitigating the formation of a sulfate layer on lead plates, a common degradation mechanism in lead–acid batteries. Simultaneously, CaO doping improves the mechanical hardness and structural stability of the electrode material, thereby limiting corrosion and extending electrode lifetime under demanding operating conditions [17,18].

Overall, studies demonstrate that doping recycled lead-based electrodes with metal oxides, such as Fe_2_O_3_, significantly enhances their thermal and electrochemical stability, thereby optimizing energy storage capacity and prolonging cycle life [18,19].

Conventional secondary lead recycling is mainly based on pyrometallurgical processing, while alternative hydrometallurgical and direct-conversion routes have been investigated to reduce energy consumption and environmental emissions and to recover lead compounds suitable for reuse as active battery materials [20]. In particular, previous studies have demonstrated the feasibility of converting spent lead paste into lead oxides that can be reused to manufacture new lead–acid battery electrodes, thereby supporting the development of more circular recycling strategies [20]. Building on this research direction, the novelty of the present study lies in the comparative investigation of melt-quenching and wet-method routes applied to spent lead–acid battery materials, and in evaluating the influence of CaO, CuO, Fe_2_O_3_, and Fe additions on the evolution of crystalline phases and electrochemical behavior. By combining two processing routes with structural and electrochemical characterization of the resulting materials, this study aims to establish relationships between recycling conditions, chemical modification, phase composition, and functional performance, with the objective of assessing their potential reuse as active electrode materials.

The aim of this study is to investigate the influence of these additives on electrode performance and to determine which combination provides the best results for real-world applications such as new electrodes in lead–acid batteries.

## 2. Materials and Methods

### 2.1. Synthesis

All oxidic systems were prepared using spent automotive battery plates as sources of PbO_2_ and Pb, along with metal oxide powders. Table 1 presents the compositions of the prepared systems, the preparation method, and the synthesis temperature.

Samples P1, P2, P3, and P4 were prepared using the conventional melt-quenching method (denoted as Method 1), a commonly employed method in glass fabrication. Depending on their composition, the starting materials consisted of CaO, CuO, Sb_2_O_3_, Fe_2_O_3_, and Fe powders, along with powder obtained from the anode (Pb source) or cathode (PbO_2_ source) of a disassembled spent automotive battery. The mixture corresponding to the desired composition was mechanically homogenized, placed in sintered corundum crucibles, and melted in air in an electric furnace at temperatures ranging from 1050 to 1150 °C, depending on the nature of the reactants. The molten material was held at this temperature for 5 or 10 min, then quenched at room temperature by pouring onto steel plates.

Samples denoted as P1N, P2N, P3N, and P4N were prepared using two methods: Method 2 (wet method) followed by Method 1. This preparation procedure is expected to yield the advanced desulfatization of the spent battery plates.

The constituent substances were weighed on an analytical balance according to the stoichiometries specified in Table 1.

The weighed powders were finely ground using an agate mortar and then transferred into a porcelain capsule. Subsequently, 3.5 mL of a 1 M NH_4_OH solution and 96.5 mL of distilled water were added to the mixture. The resulting suspension was placed on a magnetic stirrer set at 25 °C and stirred for 10 min. The temperature was increased to 75 °C for 10 min, and then to 100 °C until the liquid phase had completely evaporated. The mixture was heated to 300 °C and maintained at that temperature for 15 min, until completely dried. The resulting fine powder was melted in a corundum crucible at 1050 °C or 1100 °C, depending on the sample’s composition. After 10 min, the melt was rapidly poured between two stainless-steel plates.

**Figure 1:**
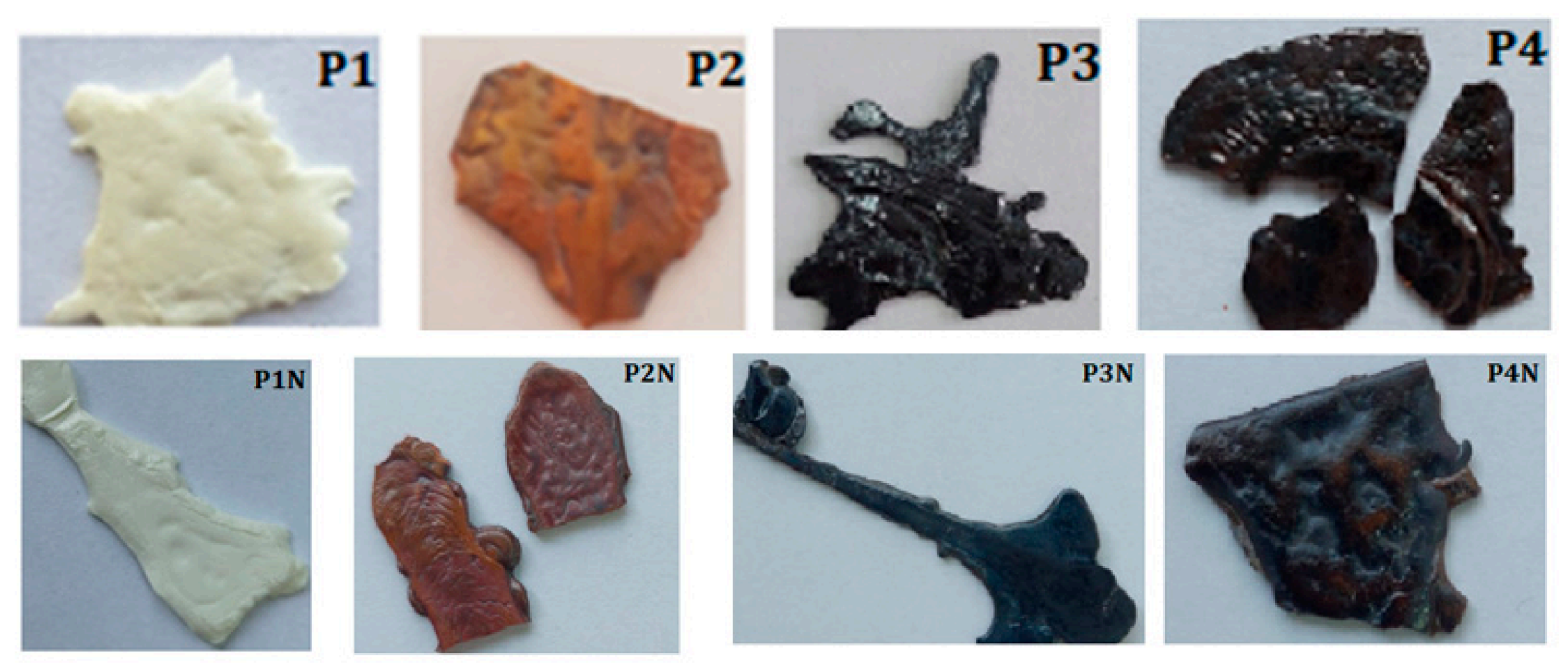
Images of samples prepared using the melt quenching method (P1, P2, P3, P4) and the wet method followed by the melt quenching method (P1N, P2N, P3N, P4N).

FujiFilm photographs of the eight samples noted with P1, P2, P3, P4, P1N, P2N, P3N, and P4N are presented in Figure 1.

As can be observed, the colors of the four systems range from white to reddish-brown and black, depending on their composition. Regarding the appearance of the samples prepared by the two methods, better homogeneity is observed in those prepared using Method 2, followed by Method 1.

### 2.2. Instruments

X-ray diffraction measurements were performed using a Shimadzu XRD-6000 diffractometer (Shimadzu Corporation, Kyoto, Japan) equipped with a graphite monochromator for a copper (*λ* = 1.54 Å) or chromium (*λ* = 2.28 Å) anode tube. The identification of crystalline phases from the diffractograms was performed using Match! software.

For the electrochemical investigations, cyclic voltammetry (CV), linear sweep voltammetry (LSV), and electrochemical impedance spectroscopy (EIS) techniques were used. Electrochemical measurements were carried out using an AUTOLAB 302N potentiostat/galvanostat (Metrohm Autolab B.V., Utrecht, The Netherlands). The instrument was equipped with a FRA32 module for impedance measurements and Nova 2 software. The NovaFit software was used to fit the EIS measurements with an adequate equivalent electrical circuit.

The experiments were carried out in an electrochemical cell with three electrodes immersed in a 5 M sulfuric acid electrolyte solution. A calomel electrode (Hg/Hg_2_Cl_2_/KCl) was used as the reference electrode, and platinum as the counter electrode. The working electrode consisted of the prepared material. A constant scan rate of 10 mV/s was employed.

## 3. Results and Discussion

### 3.1. An Analysis of the Recycling Methods of the Lead–Acid Batteries

Efficient recycling technologies are necessary to reduce environmental pollution, conserve natural resources, and improve the sustainability of lead production.

The recycling methods of lead–acid batteries can definitely differ. The goal is always the same: recovering lead, plastic, and sulfuric acid. The main differences come from how the battery is broken down and how the lead is recovered.

The pyrometallurgical recycling or smelting method is the most common traditional method. The anodic and cathodic components are melted in a furnace, and the impurities form slag, leaving purified molten lead. The method uses high temperatures (~1000 °C), efficiently handles large volumes, and produces secondary lead that can be reused in a new battery. The method is mature, uses well-established technology, and achieves a high recovery rate of over 95%. The main disadvantages of this technology are: (i) high energy consumption and (ii) the release of lead dust and emissions into the environment.

The hydrometallurgical recycling method uses chemical solutions in which lead compounds are dissolved at lower temperatures, either in acidic or alkaline media. By using chemical reactions, lead is precipitated or electrolytically recovered. As advantages, it can be noted that lower emissions than those of the pyrometallurgical process are produced, and that lead has higher purity. The disadvantages of the method are (i) more complex chemical handling, (ii) inefficient solubility, and (iii) that it is less widely used industrially.

Electrochemical recycling is a relatively new method that combines hydrometallurgy and electrolysis. In this process, in the first stage, the lead compounds are dissolved and then electrically reduced to pure lead. As a result, lead has a very high purity, and air pollution is reduced. The method is still in the commercial development stage and entails higher setup costs.

The direct recycling technology (paste-to-paste) aims to reuse lead paste from old batteries by chemically treating it without fully smelting it. Using this route results in lower emissions and lower energy consumption. The method is not yet widely used worldwide and requires advanced process control.

Table 2 lists the differences between these recycling methods.

In summary, the recycling of lead–acid batteries varies across temperature, chemical processes, energy use, environmental impact, and technological maturity.

The melt quenching method, also referred to as the melt subcooling method, is a modified thermal recycling process in which molten lead compounds are rapidly quenched after melting. During this process, the lead paste is heated to a molten state, followed by rapid subcooling that alters the melt’s crystallization behavior. The accelerated solidification promotes the separation of lead phases and limits the incorporation of impurities into the final material. The main characteristics of this method can be summarized as follows: (i) it uses controlled rapid cooling instead of prolonged smelting refinement; (ii) it modifies the crystallization pathway to enhance phase separation; and (iii) it has been proposed as a potentially more energy-efficient thermal recycling technique. Among its main advantages are shorter processing times, lower energy consumption than conventional smelting under optimized operating conditions, and reduced formation of undesirable secondary compounds. However, the process still requires precise temperature control, and its industrial implementation remains limited due to the need for specialized processing equipment.

The key differences between the process mechanisms can be described as follows: (i) for the traditional pyrometallurgical method, the chemical reduction happens at a higher temperature; (ii) the hydrometallurgical technique is characterized by dissolution and precipitation reactions; (iii) the electrochemical route is based on the electrical reduction of ions; (iv) at the melt quenching method, a physical metallurgical control of solidification is required.

The melt quenching method can be regarded as a modified pyrometallurgical approach that improves the solidification of molten lead by rapidly cooling it. Compared with conventional pyrometallurgical recycling, this method has the potential to reduce furnace residence time and energy consumption while promoting better phase separation. Despite these advantages, melt quenching is still considered an emerging technology and remains limited to laboratory and pilot-scale studies, whereas conventional pyrometallurgical recycling continues to dominate industrial lead–acid battery recycling [21].

The following sections present the structural and microstructural characterization of samples P1–P4 and P1N–P4N, providing a comparative assessment of the melt quenching and wet processing routes.

### 3.2. Analysis of X-Ray Diffraction (XRD)

**Figure 2:**
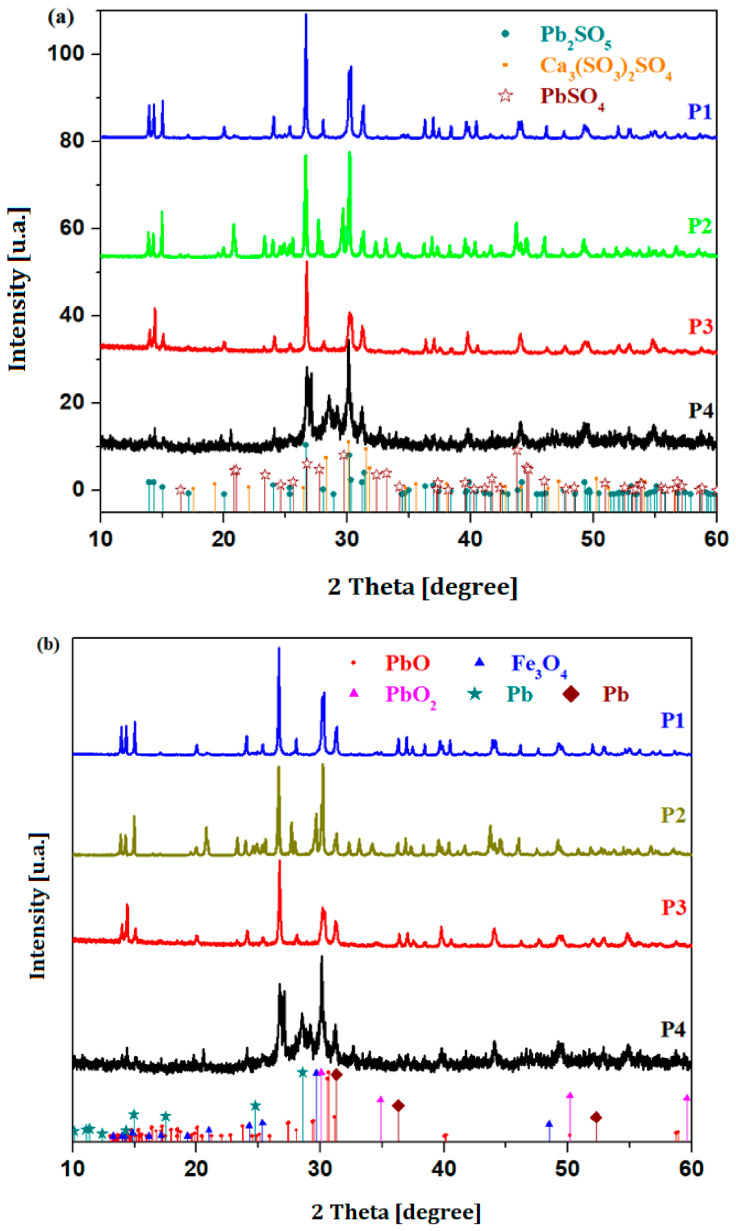

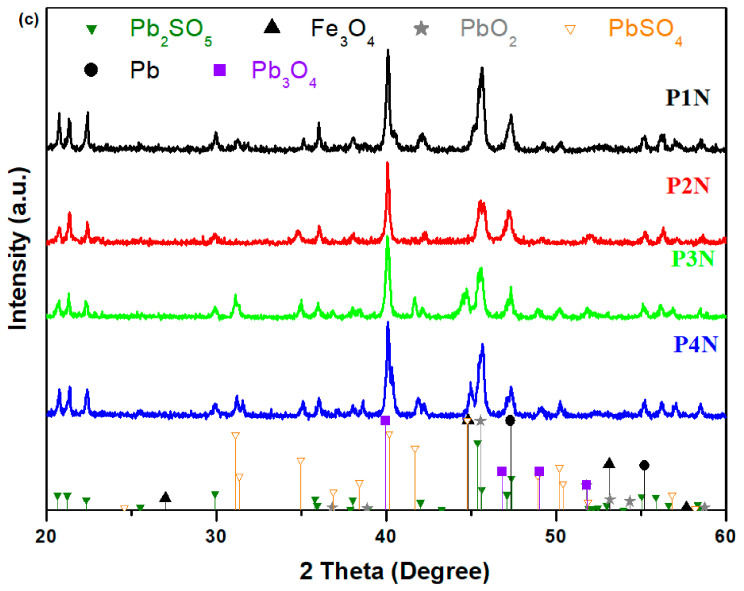
X-ray diffraction patterns of the samples: (**a**) P1, P2, P3, and P4 prepared from spent automotive battery plates and laboratory reagents using the melt undercooling method, with the identified sulfated phases; (**b**) P1, P2, P3, and P4 with the identified oxide phases; (**c**) P1N, P2N, P3N, and P4N prepared using the wet method followed by the melt undercooling method.

Figure 2 presents the XRD patterns of the prepared samples. Figure 2a,b show the XRD patterns of samples P1, P2, P3, and P4, together with the assignment of the existing crystalline phases. In all samples, the sulfated phases of lead and calcium ions were identified (see Table 3). The sample P1 has a vitroceramic structure consisting of two sulfated crystalline phases, namely Pb_2_SO_5_ ≡ PbO·PbSO_4_, and the Ca_3_(SO_3_)_2_SO_4_ crystalline phase. In the sample, P2 was detected as the crystalline phases Pb_2_SO_5_ and PbSO_4_. The sample P3 exhibits the Pb_2_SO_5_ and CaSO_4_ crystalline phases, whereas sample P4 contains only the Pb_2_SO_5_ phase. All samples contain the Pb_2_SO_5_ crystalline phase with an orthorhombic structure. This phase exhibits its main diffraction peaks at 26.7° (100% relative intensity) and 30.2° (82.5% relative intensity). It is observed that the intensities of the main diffraction peaks of this crystalline phase vary depending on the nature of the dopants. For sample P1, the diffraction peak at 26.7° has the lowest intensity, while the intensity of the second peak increases. This behavior can be attributed to the formation of calcium sulfite–sulfate phases, namely Ca_3_(SO_3_)_2_SO_4_ crystalline phase. In sample P3, the diffraction peak centered at 30.2° exhibits the lowest intensity, indicating a reduced content of the Pb_2_SO_5_ crystalline phase in this system. Samples P1 and P2 exhibit stronger diffraction peaks, suggesting a higher content of oxosulphated phases. Additionally, sample P2 contains the PbSO_4_ crystalline phase with an orthorhombic structure, whose main diffraction peak is located at 43.6°.

The analysis of the XRD patterns also reveals the presence of various phases of metallic lead, PbO, and PbO_2_. The content of the PbO_2_ crystalline phase increases in samples P2 and P4. For the sample P2 (doped with 20 mol% CuO), the peaks situated at 26.7° and 30.2° exhibit similar intensities, which suggests an increase in the PbO_2_ crystalline phase, considering that its main diffraction peak is also located around 30.2°. Sample P2 shows the highest content of metallic lead. Sample P4 contains two types of metallic lead crystalline phases, with the main diffraction peaks located at 28.4° and 31.3°. Sample P2 also exhibits the highest PbO content and contains the Fe_3_O_4_ ferrite crystalline phase with a cubic structure.

Figure 2c shows the X-ray diffractograms of the P1N, P2N, P3N, and P4N samples. XRD data evidence the presence of the Pb_2_SO_5_ crystalline phase with monoclinic structure in all samples. The presence of PbSO_4_ crystalline phase was detected in the P1N, P2N, and P4N samples. The Pb_3_O_4_, PbO_2_, and Pb crystalline phases were identified in all samples. The P3N and P4N samples contain the Fe_3_O_4_ crystalline phase in the vitroceramic matrix because Fe_2_O_3_ can be reduced to Fe_3_O_4_ at temperatures above 950 °C. The content of the Pb_2_SO_5_ crystalline phase decreases across all samples compared with their P1, P2, P3, and P4 analogs. These structural changes can be explained by the transformation of Pb_2_SO_5_ into crystalline PbSO_4_ and the simultaneous formation of crystalline Pb_3_O_4_.The presence of Fe_3_O_4_ in the host matrix can be responsible for the desulpharization process.

In conclusion, the XRD analysis indicates that the wet method promotes both the transformation of lead oxysulfate (Pb_2_SO_5_) into crystalline PbSO_4_ and the formation of crystalline Pb_3_O_4_. While the former reflects phase transformation and redistribution of sulfur-bearing species, the latter provides evidence of partial desulphatization through the formation of a sulfur-free lead oxide.

### 3.3. Voltammetric Characteristics of Electrode Materials Prepared Using the Melt Quenching Method

**Figure 3:**
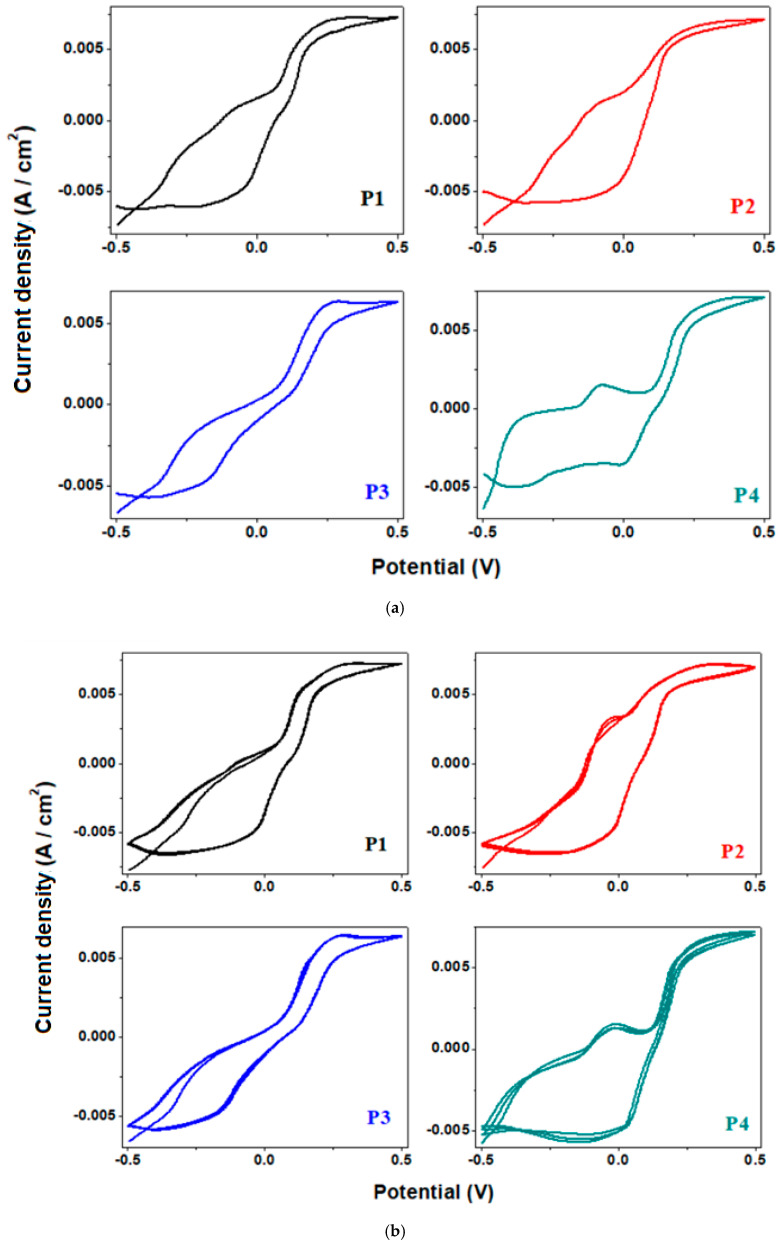
Cyclic voltammograms recorded after (**a**) one cycle and (**b**) scanning of three cycles for electrode materials P1, P2, P3, and P4.

The cyclic voltammograms recorded after the first cycle for the P1, P2, P3, and P4 samples are shown in Figure 3.

Samples P1 and P2 exhibit stable inverse hysteresis curves. The cyclic voltammograms show that the sample P2 exhibits the highest current density and the smallest peak separation, indicating superior electrochemical activity and faster electron transfer.

Sample P3 exhibits a fairly symmetric voltammogram shape, typical of a capacitive-type process with good reversibility, and a gradual increase in current between 0 and 0.5 V, without distinct oxidation or reduction peaks. Sample P3, with a smooth shape of the cyclic voltammograms, also demonstrates good reversibility with relatively high current density. Although sample P1 exhibited a slightly higher anodic peak current density (7.20 × 10^−3^ A/cm^2^) than sample P2 (7.17 × 10^−3^ A/cm^2^), the difference between the two current values was small. More importantly, sample P2 showed a lower half-wave potential (0.012 V) and lower bulk resistance (207.21 Ω) compared with sample P1 (0.043 V and 312.35 Ω, respectively), indicating a lower potential requirement for the electrochemical process and improved charge transport. Therefore, the overall electrochemical response of sample P2 suggests more favorable kinetics than that of sample P1. Sample P1 indicates moderate behavior. In contrast, sample P4 exhibits a distorted voltammogram and larger peak separation, indicating slower kinetics and increased resistance.

The cyclic voltammograms recorded after three cycles for the P1, P2, P3, and P4 samples are presented in Figure 3b. A simple inspection of the voltammograms indicates a high degree of irreversibility for the P4 sample.

**Figure 4:**
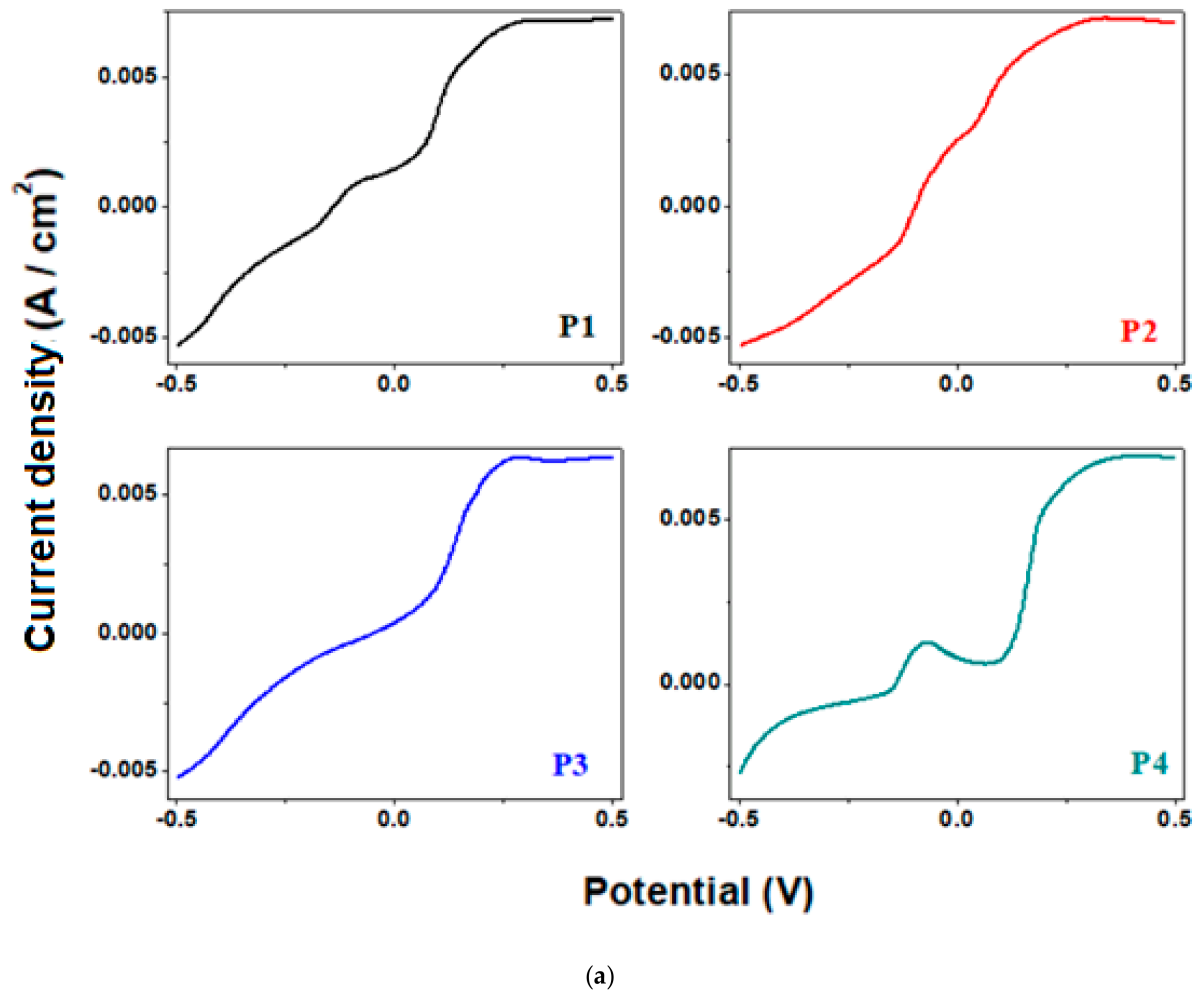

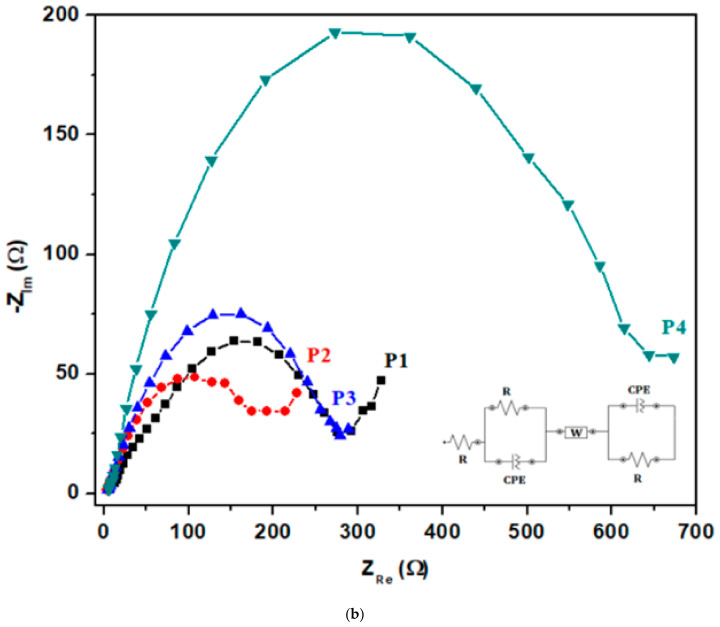
(**a**) Linear sweep voltammograms and (**b**) Nyquist diagrams for electrode materials P1, P2, P3, and P4.

The linear sweep voltammograms of the P1, P2, P3, and P4 samples are shown in Figure 4a. The electrochemical parameters derived from the linear sweep voltammograms, namely the half-wave potential (E_1/2_) after the first scan cycle, are listed in Table 4.

The half-wave potential indicates the potential at which the electrochemical reaction occurs, while the anodic current density reflects the rate of the electrochemical reaction. The resistance parameter is associated with the material’s charge-transfer properties and electrical conductivity. By comparing these parameters among varied samples, it is possible to determine the electrochemical efficiency and suitability of recycled lead materials for reuse.

The half-wave potential values (E_1/2_) increase in the following ascending order: P3 < P2 < P1 < P4. The lowest half-wave potential values were obtained for the P3 and P2 samples, while, as expected, the P1 and P4 samples exhibit the highest E_1/2_ values. Doping with 30% CaO appears to reduce conductivity and electrochemical activity, possibly due to the formation of the Ca_3_(SO_3_)_2_SO_4_ crystalline phase. In the case of the P2 sample, CuO doping makes a significant contribution to redox activity, which explains the unstable yet highly active CV curves. For the P3 sample, Fe doping introduces redox-active centers, resulting in higher intensity and capacitive behavior suitable for long-life energy storage systems. Sample P4, containing 3% CaO, exhibits instability and a highly irreversible voltammogram.

In conclusion, the P1 and P2 samples exhibit the highest anodic current density (7.21 × 10^−3^–7.23 × 10^−3^ A/cm^2^), indicating the highest electrochemical activity. The P3 sample has the lowest half-wave potential, indicating good reversibility of the voltamogram. The P4 sample has the lowest current density and the highest half-wave potential, indicating weaker activity and the highest grade of irreversibility in the cyclic voltammogram.

The half-wave potential varies widely, which can be associated with differences in reaction kinetics or composition. The best performances are found for the P2 and P3 samples.

Nicholson et al. [22] reported that the shape and magnitude of anodic and cathodic peaks can indicate whether a reaction is reversible or irreversible. When anodic and cathodic peak values are close, the redox reaction is relatively fast and exhibits good reversibility. Laviron [23] described how electrochemical behavior changes in systems dominated by surface-controlled processes rather than purely diffusion-controlled ones. In such cases, even background currents become significant, as they may indicate parasitic reactions.

In practical applications, current density is commonly used as a performance criterion. Wang [24] highlighted that higher peak currents may indicate larger active surface areas or improved conductivity. A concrete example is the study by Mirzaeian et al. [25] on carbon electrodes for supercapacitors, which demonstrated that pore structure directly influences the current density values and, consequently, energy storage capacity.

In conclusion, current density is not merely a normalized value but rather represents a “signature” of electrode behavior, reflecting its activity, stability, and efficiency within an electrochemical system. The overall ranking based on CV, LSC, and EIS performances for electrochemical activity and kinetics decreases from sample P2 > sample P3 > sample P1 > sample P4.

### 3.4. Voltammetric Studies of Materials Prepared by a Complex Method

**Figure 5:**
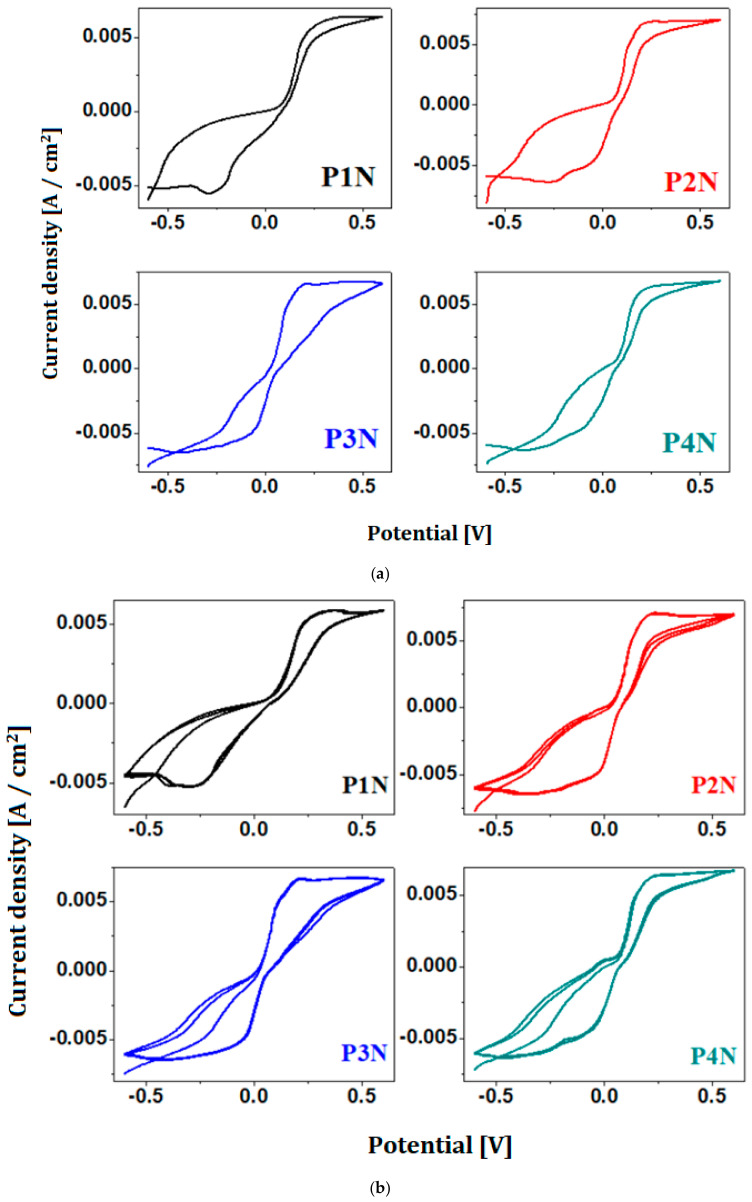
Cyclic voltammograms recorded after scanning of: (**a**) the first cycle and (**b**) three cycles of samples P1N, P2N, P3N, and P4N.

The cyclic voltammograms of the P1N, P2N, P3N, and P4N samples are presented in Figure 5a. All samples exhibit symmetric cyclic voltammetry curves with a general “loop-like” shape, characteristic of reversible or quasi-reversible processes. All samples exhibit asymmetric cyclic voltammetric profiles characterized by pronounced cathodic current responses and broad reduction features in the negative potential region, with differences in profile shape and current response reflecting the influence of sample composition on the electrochemical behavior. Compared to the cyclic voltammograms of the P1, P3, and P4 samples, the reduction waves are more clearly defined.

Figure 5b shows the cyclic voltammograms recorded after three cycles for the P1N, P2N, P3N, and P4N samples. Sample P1N exhibits relatively stable curves between cycles, with a slight increase in current density at the potential limits compared to the first cycle. The overall shape remains symmetrical, without distinct redox peaks.

Sample P2N shows a slight increase in current density with increasing cycle number, which may suggest the gradual activation of electrochemically active centers or an improvement in electrode-electrolyte contact.

Sample P3N displays the widest voltammetric loop and a more pronounced difference between cycles, indicating progressive activation of the material or slight surface restructuring.

Sample P4N exhibits consistent behavior, with good overlap between cycles, indicating a stable active structure.

**Figure 6:**
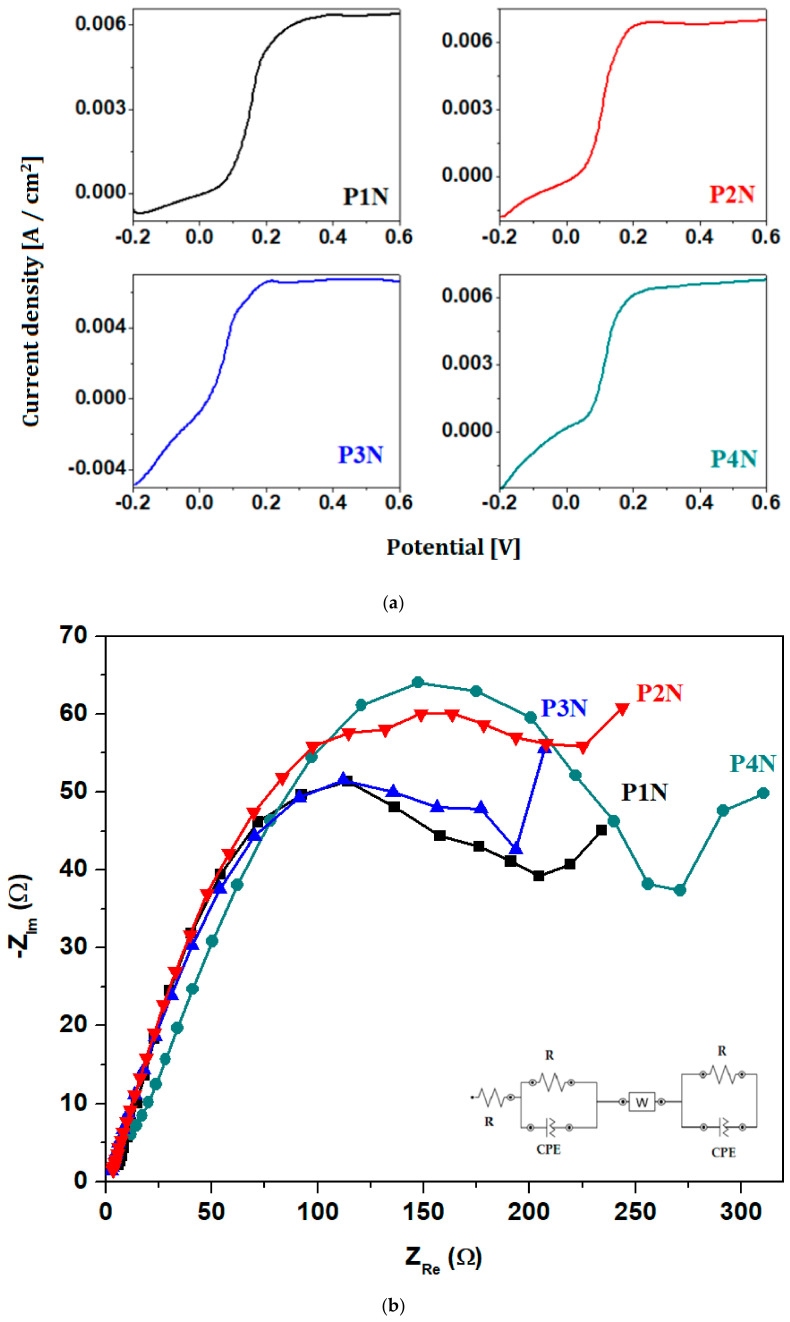
(**a**) Linear sweep voltammograms and (**b**) Nyquist plots of the complex impedance of samples P1N–P4N.

Figure 6a presents the linear sweep voltammograms of P1N–P4N samples, which indicate behavior typical of materials with a stabilized passive layer [26].

Figure 6b shows the electrochemical impedance spectra of P1N, P2N, P3N, and P4N samples, together with the equivalent circuit used for electrochemical simulation. The impedance spectra consist of a semicircle in the high-frequency region, followed by a pronounced, inclined linear segment in the low-frequency region. The Nyquist plots reveal clear differences between the semicircles corresponding to each sample. Sample P3N exhibits the smallest semicircle, while sample P4N shows a larger semicircle and a more pronounced Warburg tail, characteristic of additional diffusion limitations.

Charge-transfer resistance, R_ct_, is determined by identifying the point of intersection between the semicircle and the *x*-axis in the low-frequency region. The electrical conductivity of the investigated electrolyte solution is inversely proportional to this resistance.

We can compare the samples by examining how the half-wave potential, anodic current density, and resistance vary among them. These parameters usually describe the electrochemical behavior of materials recovered from a lead–acid battery recycling process.

The equivalent circuit model [R(RQ)W(RQ)] consists of solution resistance, constant phase elements (CPE), and a Warburg element that accounts for diffusion. In the low-frequency region, the Nyquist plots exhibit an inclined linear response associated with diffusion-controlled impedance behavior, represented by the Warburg element (W) in the equivalent circuit model [27]. In the medium-frequency region, the appearance of a semicircle reflects the charge-transfer resistance. Differences in the arc diameters indicate variations in interfacial charge-transfer kinetics.

The electrochemical parameter values for the P1N–P4N samples are presented in Table 5 and show significant differences among the materials. The P3N sample exhibited the highest electrical conductivity because it had the lowest resistance.

The electrochemical parameters summarized in Table 5 reveal distinct responses among the wet-processed samples. Although P2N exhibits the highest anodic peak current density, P3N shows the lowest charge-transfer resistance value (R_ct_), indicating a reduced resistive contribution to the overall electrochemical response. In contrast, P4N exhibits the highest R_ct_, suggesting greater resistance to the electrochemical processes occurring within the system. Therefore, the anodic peak current density and resistance values describe different aspects of the electrochemical behavior, and the favorable overall response of P3N is associated with its lower resistance, combined with a relatively high anodic current density.

The lowest half-wave potential (E_1/2_ = 0.07 V) was observed for the P3N sample, indicating a higher thermodynamic affinity for the redox reaction. This trend is further supported by the charge-transfer resistance R_ct_, which is also lowest for this sample (194.63 Ω), indicating faster charge-transfer kinetics. At the opposite extreme, the P4N sample shows a higher E_1/2_ of 0.11 V and the highest charge-transfer resistance (263.20 Ω), confirming that the redox reaction is less favorable and proceeds more slowly.

This correlation between the half-wave potential (E_1/2_) and the resistance (R_ct_) values indicates that the electrode material’s structure influences both the thermodynamic and kinetic characteristics of the electrochemical reaction.

**Figure 7:**
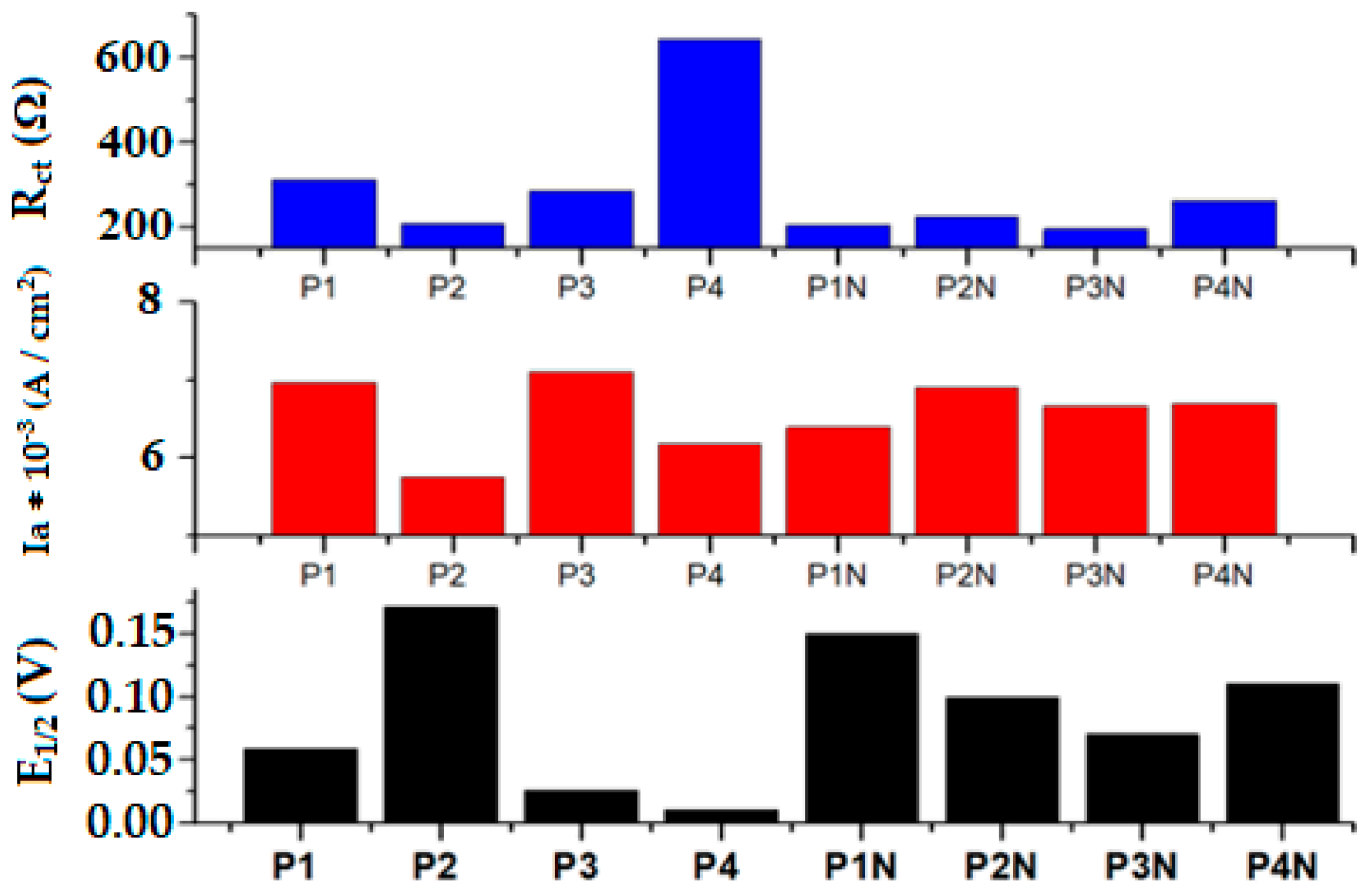
The compositional evolution of electrochemical parameters.

Figure 7 shows the compositional evolution of the electrochemical parameters, including the half-wave potential and anodic intensity, for the samples studied. The lower values of the half-wave potential are denoted for P1, P3, P4, and P3N. The highest current densities are observed in the P1, P3, P2N, P3N, and P4N samples. A comparative study regarding these electrochemical parameters suggests that the P3 and P3N samples show better performance than their analogs. Additionally, the P2N sample shows improved parameters (the value of E_1/2_ decreases and the value of I_a_ increases) upon complex preparation compared with the P2 sample.

In conclusion, the P3 and P3n samples have better electrochemical properties. Higher I_a_ and lower R_ct_ values generally indicate better charge transfer and conductivity, which is beneficial for materials obtained during the lead recovery process.

The electrochemical parameters of the samples show noticeable differences in half-wave potential and current density. In the first dataset, the P3 sample shows the highest anodic current density, suggesting superior electrochemical activity. In the second dataset, the P3N sample indicates noticeable differences in half-wave potential and current density. These results indicate that the P3N and P3 samples exhibit the most favorable electrochemical performance among the materials studied.

The addition of CaO, Fe_2_O_3_, and Fe to the spent active mass of anodic plate (considered as a Pb source) from a lead–acid battery shows superior electrochemical performances compared to the other compositions when it is prepared directly by the melt quenching method or by the complex method, respectively.

By adding CuO and Sb_2_O_3_ to the PbO_2_–Pb host matrix derived from the spent cathodic and anodic masses of a lead–acid battery, the half-wave potential and current density can be optimized, making it suitable for use as a new electrode when prepared via a complex method.

### 3.5. Differences Between the Two Groups of Samples

To estimate which method is more efficient, we compare the XRD diffractograms and electrochemical parameters of the samples prepared by the melt quenching method (P1–P4 samples) and the complex method (P1N–P4N) for vitroceramics obtained from a lead–acid battery by the recycling process.

- Comparison of structure

The presence of crystalline phases indicates the vitroceramic structure of all samples. To compare the groups of samples P1–P4 and P1N–P4N using XRD phase identification results, we can assess them in terms of phase composition, oxidation state of lead, and possible transformation processes.

XRD analysis showed that both groups contain crystalline Pb, PbO_2_, and Pb_2_SO_5_ phases, indicating the persistence of common lead-containing phases after processing. Group 1 (P1–P4) also contains PbO and calcium-bearing sulfur compounds, with Ca_3_(SO_4_)(SO_3_)_2_ identified in P1 and CaSO_4_ in P3. In Group 2 (P1N–P4N), additional PbSO_4_ and Pb_3_O_4_ phases were identified, while Fe_3_O_4_ was detected in P3N and P4N. PbSO_4_ was observed in P1N, P2N, and P4N, with a lower contribution of the Pb_2_SO_5_ phase than in Group 1.

These differences indicate that the two processing routes promote distinct pathways for phase evolution. The presence of PbO and calcium-containing sulfate/sulfite phases in the melt-quenched samples suggests the retention of oxide phases and sulfur-containing calcium compounds under the applied processing conditions. In contrast, the wet-processed samples exhibit redistribution of the sulfur-bearing lead phases, as indicated by the lower contribution of Pb_2_SO_5_ and the formation of crystalline PbSO_4_. This transformation should be interpreted as phase conversion or redistribution of sulfur-bearing species rather than desulphatization. The occurrence of Pb_3_O_4_ further indicates the formation of a mixed-valence lead oxide phase, whereas Fe_3_O_4_ in P3N and P4N confirms the incorporation of iron-containing crystalline phases. Overall, the XRD results demonstrate that the wet processing route modifies the phase assemblage of the lead-containing materials and promotes the formation of PbSO_4_, Pb_3_O_4_, and, in selected samples, Fe_3_O_4_ compared with the melt-quenching route.

- Comparison of electrochemical parameters

The samples P1, P2, P3, and P4, prepared by the melt quenching method, can be distinguished from the samples P1N, P2N, P3N, and P4N, prepared by the complex method, based on their electrochemical parameters (see Table 6).

In the first group (P1–P4), the values of the half-wave potential range from 0.012 to 0.137 V, the anodic current density ranges from 6.31 × 10^−3^ to 7.20 × 10^−3^ A/cm^2^, and the bulk resistance increases from 207.21 to 642.59 Ω. Within this group, samples P2 and P3 exhibit higher electrochemical activity/kinetics, as well as a larger capacitive area. In contrast, sample P4 shows poor reversibility, slow kinetics, the lowest electrochemical activity, a moderate peak current, and a medium capacitive area.

In the second group (P1N–P4N), the half-wave potential ranges between 0.07 and 0.15 V, the anodic current density ranges between 6.397 × 10^−3^ and 11 × 10^−3^ A/cm^2^, and the bulk resistance ranges between 194.63 and 263.2 Ω. Among these samples, P3N exhibits the lowest resistance at relatively high current density, indicating improved electrical conductivity and better electrochemical performance compared with the other samples in this group.

The results indicate that the preparation method may influence the electrical conductivity and charge-transfer properties of the materials.

The complex method samples (P1N–P4N) generally exhibit more stable electrochemical behavior and measurable resistance value, indicating better control of electrical properties. In contrast, the samples P1–P4 show greater variation in current density and half-wave potential, which may be related to differences in doping levels.

Therefore, the samples prepared by the complex method can be clearly differentiated from the samples P1–P4 through their electrochemical parameters. The results suggest that the complex preparation method and the nature of the dopant type influence the conductivity, resistance, and electrochemical activity of the recycled lead materials derived from lead–acid battery recycling.

In conclusion, both preparation methods show good electrochemical behavior (except for the sample P4). The charge-transfer resistance of the P4 sample was decreased when the sample was prepared by the wet/melt quenching method. However, the samples P2 and P3 synthesized by the melt quenching method have higher electrochemical activity. On the other hand, the complex method yields more consistent electrochemical properties and lower charge-transfer resistance, indicating better control of the material structure, consistent with XRD analysis. The doping with CaO, Fe_2_O_3_, and Fe in the sample P3N of the spent lead electrode exhibits superior electrical conductivity and faster redox kinetics, as confirmed by the lowest values of the resistance R_ct_ and the half-wave potential E_1/2_.

Based on CV, LSV, and EIS analyses, samples P2 and P3N demonstrate the highest electrochemical performance for battery applications, exhibiting the largest closed area and the smallest peak separation, indicating high capacity and fast charge-transfer kinetics. Samples P3 and P4N also show promising behavior with good reversibility and relatively high current response. The samples P1 and P2N exhibit moderate performances. Samples P4 and P1N exhibit poor electrochemical performance due to sluggish kinetics and low reversibility, making them less suitable for battery applications.

## 4. Conclusions

A set of eight samples were prepared using two methods: the melt quenching method and a more complex route that combines the wet method with melt quenching.

It can be concluded that the complex preparation method generates combinations of lead oxides and lead sulfate phases, with their relative balance being highly sensitive to the control of processing conditions.

The cyclic voltammetry results show clearly that the nature of the dopant influences both the stability and the reversibility of the redox processes. The P1, P2, and P3 samples exhibit more stable and reversible behavior, whereas the P4 sample, although electrochemically active, shows a high degree of irreversibility due to secondary reactions. The current density, half-wave potential, and charge-transfer resistance prove to be essential indicators for evaluating the electrochemical performances of the recycled electrode materials.

The combined results of cyclic voltammetry and electrochemical impedance spectroscopy demonstrate that the P3N sample exhibits a superior electrical conductivity and faster redox kinetics, as confirmed by the lowest values of the resistance R_ct_ and the half-wave potential E_1/2_. In contrast, the P1N sample clearly shows the diffusion limitations and reduced redox activity. These observations highlight the direct influence of material composition and structure on electrochemical stability and performance.

The samples P3 (doped with Fe) and P4N (CaO) demonstrate good reversibility and stability. In contrast, the samples P1 (doped with CaO) and P2N (doped with CuO) evidence moderate activity. While the sample P4 (CaO) exhibits significant polarization and distorted redox behavior, likely due to sulfation effects, it is less suitable for practical applications. The sample doped with CaO, Fe_2_O_3_, and Fe exhibits the lowest resistance, along with a relatively high current density, suggesting improved electrical conductivity and favorable charge-transfer characteristics. Overall, the wet-assisted processing route generally improved the electrochemical characteristics of the recycled lead-based materials. However, the most favorable electrochemical performance was observed for samples P2N and P3N, while the remaining compositions exhibited varying degrees of improvement depending on the evaluated electrochemical parameter.

The quenching method indicates a wider variation of half-wave potential, peak current, and, charge-transfer resistance, which can be attributed to less uniformity between samples.

Therefore, the quenching method may provide higher peak electrochemical activity, while the wet/quenching method appears to offer more stable and uniform electrochemical performance for recycled lead materials from lead–acid battery processing.

## Figures and Tables

**Table 1:** Composition of the prepared systems, synthesis methods, and operating temperatures.

Sample	Compozition	Methodes	Temperature
**P1**	xCaO·(100 − x) [4PbO_2_·Pb], x = 30% CaO	Method 1	1050 °C
**P1N**		Method 2 + Method 1	25–300 °C + 1050 °C
**P2**	xCuO·10Sb_2_O_3_·(90 − x) [4PbO_2_·Pb], x = 20% CuO	Method 1	1050 °C
**P2N**		Method 2 + Method 1	25–300 °C + 1050 °C
**P3**	3CaO·5Fe_2_O_3_·xFe·(92 − x)Pb, x = 8% Fe	Method 1	1150 °C
**P3N**		Method 2 + Method 1	25–300 °C + 1150 °C
**P4**	xCaO·5Fe_2_O_3_·(95 − x)Pb, x = 3% CaO	Method 1	1050 °C
**P4N**		Method 2 + Method 1	25–300 °C + 1050 °C

**Table 2:** Comparison of recycling methods based on operating temperature, chemical processes, and technological maturity.

Method	Temperature	Main Technique	Maturity
Pyrometallurgical	high	smelting	very common
Hydrometallurgical	low	chemical leaching	growing
Electrochemical	low	electrolysis	emerging
Direct recycling	low	paste reuse	experimental

**Table 3:** Identification of the crystalline phase in prepared vitroceramics.

Group	Sample	Identification Phase	Observation
1	P1	Pb, PbO, PbO_2_, Pb_2_SO_5_, Ca_3_(SO_3_)_2_(SO_4_)	
	P2	Pb, PbO, PbO_2_, Pb_2_SO_5_, PbSO_4_, Fe_3_O_4_	
	P3	Pb, PbO, PbO_2_, Pb_2_SO_5_, CaSO_4_	Lowest Pb_2_SO_5_
	P4	Pb, PbO, PbO_2_, Pb_2_SO_5_	
2	P1N	Pb, PbO_2_, Pb_3_O_4_, Pb_2_SO_5_, PbSO_4_	The content of Pb_2_SO_5_ is reduced compared with group 1
	P2N	Pb, PbO_2_, Pb_3_O_4_, Pb_2_SO_5_, PbSO_4_	
	P3N	Pb, PbO_2_, Pb_3_O_4_, Pb_2_SO_5_, Fe_3_O_4_	
	P4N	Pb, PbO_2_, Pb_3_O_4_, Pb_2_SO_5_, PbSO_4_, Fe_3_O_4_	

**Table 4:** Electrochemical parameters of the prepared materials, namely the half-wave potential, E_1/2_, anodic current intensity, I_a_, and charge-transfer resistance, R_ct_, for group 1.

Electrode	x Mole %	E_1/2_	I_a_ × 10^−3^ (A/cm^2^)	R_ct_ (Ω)	Interpretation
**P1**	**30% CaO**	0.043	7.20 (highest)	312.35	High current density, moderate resistance
**P2**	**20% CuO**	0.012 (lowest)	7.17	207.21 (lowest)	Best electrochemical activity and better charge transfer
**P3**	**8% Fe**	0.078	6.31 (lowest)	286.6	Moderate activity and resistance
**P4**	**3% CaO**	0.137 (highest)	6.92	642.59 (highest)	Lower electrochemical activity, highest resistance
Charge-transfer efficiency decreases ∗ Sample P2 > Sample P3 > Sample P1 > Sample P4					
The apparent electron-transfer performance ∗∗ Sample P2 > Sample P3 > Sample P1 > Sample P4					

∗ This hierarchy is based primarily on the lower half-wave potential and lower bulk resistance values, rather than on the anodic peak current density alone. ∗∗ Although sample P1 exhibits the highest anodic peak current density, sample P2 shows the lowest half-wave potential and bulk resistance. Therefore, the sequence P2 > P3 > P1 > P4 should be interpreted as an overall charge-transfer efficiency ranking, not as a ranking based solely on anodic current magnitude.

**Table 5:** Electrochemical parameters of electrode materials P1N, P2N, P3N, and P4N.

Electrode	x Mole %	E_1/2_ (V)	I_a_ × 10^−3^ (A/cm^2^)	R_ct_ (Ω)	Interpretation
**P1N**	**30% CaO**	0.15 (highest)	6.39 (lowest)	204.32	Moderate current, moderate resistance
**P2N**	**20% CuO**	0.10	6.89 (highest)	224.95	Higher current but moderate resistance
**P3N**	**8% Fe**	0.07 (lowest)	6.67	194.63 (lowest)	Good conductivity Better charge transfer
**P4N**	**3% CaO**	0.11	6.70	263.20 (highest)	Highest resistance Poorer conductivity
Charge-transfer efficiency decreases: Sample P3N > Sample P1N > Sample P2N > Sample P4N					
The apparent electron-transfer performance: Sample P3N > Sample P1N > Sample P2N > Sample P4N					

**Table 6:** Overall evolution of the electrochemical parameters for groups 1 and 2.

Criterion	Quenching Method (P1–P4)	Complex Method (P1N–P4N)
Current density (I_a_)	6.31–7.20 A/cm^2^ Highest value observed	6.39–6.89 A/cm^2^ High and stable
Half-wave potential, E_1/2_	0.012–0.137 V Larger variation	0.07–0.15 V Moderate variation
Resistance, R_ct_	207.21–642.59 Ω Highest value observed	194.63–263.2 Ω Measured and low
Uniformity	Larger variation	More consistent

## Data Availability

The original contributions presented in this study are included in the article. Further inquiries can be directed to the corresponding author.

## References

[1] Rada S., Cuibus D., Vermesan H., Rada M., Culea E. (2018). Structural and Electrochemical Properties of Recycled Active Electrodes from Spent Lead-Acid Battery and Modified with Different Manganese Dioxide Contents. Electrochim. Acta.

[2] Piscoiu D.N., Rada S., Macavei S., Popa A., Crisa C.A., Vermesan H., Culea E. (2024). The Effect of Calcium and Iron (III) Oxides on Lead Spent Plates: Spectroscopic, Voltametric, and EIS Investigations. Materials.

[3] Piscoiu D.N., Rada S., Vermesan H. (2025). Enhancing electrochemical properties of vitreous materials based on CaO–Fe_2_O_3_–Fe–Pb and recycled from anodic plate of a spent car battery. Materials.

[4] Rada S., Unguresan M., Rada M., Tudoran C., Wang J., Culea E. (2020). Performance of the Recycled and Copper-Doped Materials from Spent Electrodes by XPS and Voltammetric Characteristics. J. Electrochem. Soc..

[5] Piscoiu D.N., Rada S., Macavei S., Barbu L., Suciu R., Culea E. (2025). Recycling of Lead-Acid Battery Electrodes Using Sb_2_O_3_ and CuO: Characterization and Electrochemical Investigations. Materials.

[6] Piscoiu D.N., Rada S., Macavei S., Vermesan H., Culea E. (2022). Characterization of Calcium Oxide Treated Lead–Lead Dioxide Vitroceramics from Recycled Automobile Batteries by X-Ray Diffraction, Infrared and Ultraviolet–Visible Spectroscopy, and Voltammetry. Anal. Lett..

[7] Ponrouch A., Bitenc J., Dominko R., Lindahl N., Johansson P., Palacin M.R. (2019). Multivalent rechargeable batteries. Energy Storage Mater..

[8] Padigi P., Goncher G., Evans D., Solanki R. (2015). Potassium barium hexacyanoferrate—A potential cathode material for rechargeable calcium ion batteries. J. Power Sources.

[9] Lipson A., Han S.-D., Kim S., Pan B., Sa N., Liao C., Fister T., Burrell A., Vaughey J., Ingram B. (2016). Nickel hexacyanoferrate, a versatile intercalation host for divalent ions from nonaqueous electrolytes. J. Power Sources.

[10] Tojo T., Sugiura Y., Inada R., Sakurai Y. (2016). Reversible Calcium Ion Batteries Using a Dehydrated Prussian Blue Analogue Cathode. Electrochim. Acta.

[11] Luo Y., Qixi Z., Ao S., Muyi S., Dianchen X., Yan Y. (2022). Calcium-doping effects on structure and electric performances of garnet-type Li_6.6_La_3_Zr_1.6_Sb_0.4_O_12_ solid-state electrolytes. Solid State Ion..

[12] Martinez-Cisneros C.S., Fernandez A., Antonelli C., Levenfeld B., Varez A., Vezzù K., Noto D., Sanchez J.-Y. (2020). Opening the door to liquid-free polymer electrolytes for calcium batteries. Electrochim. Acta.

[13] Gudkov S.V., Burmistrov D.E., Fomina P.A., Validov S.Z., Kozlov V.A. (2024). Antibacterial Properties of Copper Oxide Nanoparticles (Review). Int. J. Mol. Sci..

[14] Devaraji M., Thanikachalam P.V., Elumalai K. (2024). The potential of copper oxide nanoparticles in nanomedicine: A comprehensive review. Biotechnol. Notes.

[15] Rydosz A. (2018). The Use of Copper Oxide Thin Films in Gas-Sensing Applications. Coatings.

[16] Diachenko O., Kováč J., Dobrozhan J., Novák O., Kováč P., Skriniarova J., Opanasyuk J. (2021). Structural and Optical Properties of CuO Thin Films Synthesized Using Spray Pyrolysis Method. Coatings.

[17] Dzenzerskii V.A., Bashev V.F., Tarasov S.V., Ivanov V.A., Kostina A.A., Korpach S.V. (2014). Structure and properties of a Pb-Ca-Sn battery alloy crystallized under nonequilibrium conditions. Inorg. Mater..

[18] Hao Z.D., Xu X.L., Liu J.B., Yan H. (2018). Review on the roles of carbon materials in lead-carbon batteries. Ionics.

[19] Singh R.K., Srinivasan A. (2009). EPR and magnetic properties of MgO–CaO–SiO_2_–P_2_O_5_–CaF_2_–Fe_2_O_3_ glass–ceramics. J. Magn. Magn. Mater..

[20] Zhang W., Yang J., Wu X., Hu Y., Yu W., Wang J., Dong J., Li M., Liang S., Hu J. (2016). A Critical Review on Secondary Lead Recycling Technology and Its Prospect. Renew. Sustain. Energy Rev..

[21] Wang H., Chen X., Cai M., Wang S., Zhou F., Wu Y., Wang D., Wang H., Wang B., Pang F. (2026). Recycling of lead-acid batteries: A review. Energy Storage Mater..

[22] Nicholson R.S., Shain I. (1964). Theory of Stationary Electrode Polarography: Single Scan and Cyclic Methods Applied to Reversible, Irreversible, and Kinetic Systems. Anal. Chem..

[23] Laviron E. (1979). General Expression of the Linear Potential Sweep Voltammogram in the Case of Diffusionless Electrochemical Systems. J. Electroanal. Chem..

[24] Wang J. (2006). Analytical Electrochemistry.

[25] Mirzaeian M., Hall P.J. (2009). Preparation of controlled porosity carbon aerogels for energy storage in rechargeable lithium oxygen batteries. Electrochim. Acta.

[26] Varela F.E., Vela M.E., Vilche J.R., Arvia A.J. (1993). Kinetics and mechanism of PbSO_4_ electroformation on Pb electrodes in H_2_SO_4_ aqueous solutions. Electrochim. Acta.

[27] Basics of Electrochemical Impedance Spectroscopy. https://www.gamry.com/application-notes/EIS/basics-of-electrochemical-impedance-spectroscopy.

